# Pachydermodactyly, mimicker of rheumatoid hands, presents in a patient with Tuberous Sclerosis

**DOI:** 10.12669/pjms.39.2.6631

**Published:** 2023

**Authors:** Halla Tariq, Aroosha Ihsan, Asadullah Khan, Roshila Shamim

**Affiliations:** 1 Dr. Halla Tariq, MBBS., 807 Eucalyptus Court Lodi California 95242, US; 2 Dr. Aroosha Ihsan, MBBS., Anatomy Lecturer, Wah Medical College, Pakistan; 3 Dr. Asadullah Khan, FCPS (Medicine), FCPS, MRCP, SCE (Rheumatology)., Assistant Professor and Head of Rheumatology, Bolan Medical College, Quetta, Pakistan; 4 Dr. Roshila Shamim, FCPS (Medicine), FCPS (Rheumatology). Karachi, Pakistan

**Keywords:** Pachydermodactyly, Tuberous sclerosis, Rheumatoid arthritis, Joint deformities

## Abstract

Pachydermodactyly (PDD) is a rare benign condition characterized by painless soft tissue swelling of small joints of hands. The most common presentation is bilateral Symmetrical swelling of proximal interphalangeal and metacarpophalangeal joint similar to Rheumatoid arthritis. The etiology of this disease is unknown, and it sometimes can coexist with other diseases. We present here a case of PDD coexisting with Tuberous Sclerosis, an autosomal dominant genetic disorder characterized by of formation of multiple benign multisystem tumors.

## INTRODUCTION

Pachydermodactyly (PDD) is a rare benign variant of digital fibromatosis that results from abnormal collagen deposition in the periarticular dermis and leads to swellings present around the proximal interphalangeal joints (PIP) and metacarpophalangeal joints (MCP).^1^ The disease was first described in 1973, by Bazex, and the term Pachydermodactyly (Pachy: thick, dermis: skin, and dactylos: fingers) was coined by Verbov in 1975.^2^,^3^ Although, clinical features of this disease mimics inflammatory arthritis, but these swellings don’t have any inflammatory features, nor cause any functional limitation. PDD can rarely occur in association with other diseases such as Ehlers- Danlos Syndrome, and Tuberous Sclerosis. PPD due to its symmetrical involvement of hand joints is a close differential of Inflammatory Arthritides. We present a case of Pachydermodactyly with concurrent Tuberous sclerosis.

## CASE PRESENTATION

A 21-year-old male form Afghanistan, known case of tuberous sclerosis, diagnosed since childhood, presented to the rheumatology clinic with the complaint of painless swellings of his finger joints with some limitation for five years. The symptoms had insidious onset, progressively increasing in size, involving both hands, and feet joints. There was no pain, or any other associated features such as fever, rash and discharge on overlying skin. Patient did notice some restriction to joint movement at full flexion only. His review of symptoms was negative for any features of Inflammatory joint pain. The patient found his swellings cumbersome not only due to limitation of joints but for cosmetic reasons as well. Patient was undergoing treatment for Tuberous Sclerosis manifesting with multiple angiofibroma for the past 3-4 years but hasn’t had any significant improvement. His past medical history was insignificant for seizures, headaches, cardiac, or other neurological symptoms. Family history is negative for Tuberous Sclerosis.

**Fig.1:**
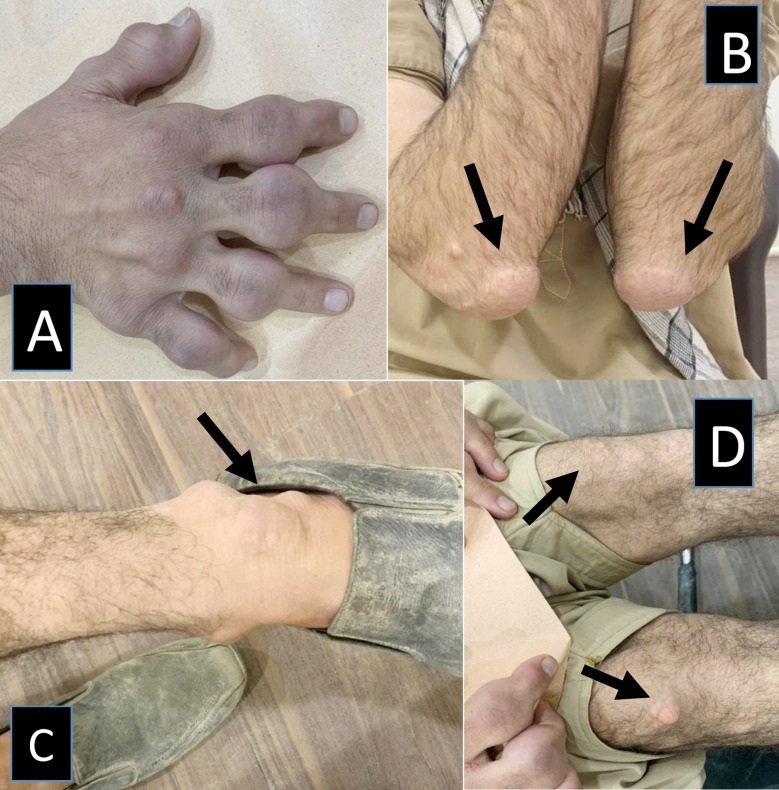
Shows swelling involving multiple joints (A) symmetrical swellings on both lateral sides of multiple metacarpophalangeal joints (MCP), proximal interphalangeal (PIP) joints, (B), (C), (D) Xanthomas, angiofibroma of tuberous sclerosis.

Upon Clinical examination; His vital signs were within normal limits for age. Patient had symmetrical swellings on both lateral sides of multiple metacarpophalangeal joints (MCP), proximal interphalangeal (PIP) joints, and metatarsophalangeal (MTP) joints. Swellings were non tender, non-fluctuating, of hard consistency, with no adherence to either overlying skin or underling bone or joint structure, and were freely mobile on hand examination. The swellings characteristically were present only on the lateral aspects of the joints line, sparing anterior and posterior aspects of joints (Fig.1A). Patient also had xanthelasma over the upper eyelids, and multiple angiofibroma over elbows, knee and ankle joint consistent with findings of Tuberous sclerosis (Fig.1: B, C, D). The rest of the physical examination was normal.

**Fig.2:**
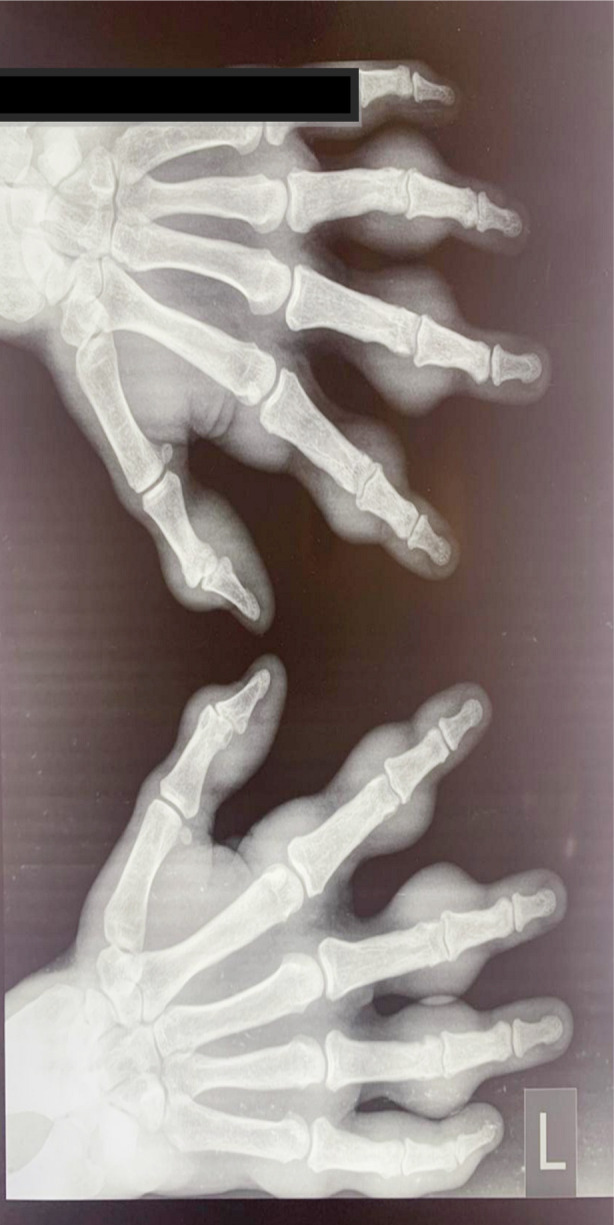
X-ray hands showing soft tissue swellings around the joint with no bony erosion

His laboratory investigations (hematological, renal and liver enzymes) revealed no abnormalities. Serum Lipid profile showed dyslipidemia, whereas uric acid levels were normal. His Rheumatoid factor, anti-CCP antibodies, anti-nuclear antibodies (ANA), were all negative. His X-ray hands revealed soft tissue swellings around the joint with no bony erosions or any joint involvement around the MCPs and PIPs (Fig.2). Screening for the cardiac anomaly was done by Echocardiography which showed normal heart structure and Ejection fraction. Brain assessment for Tuberous sclerosis by MRI Brain depicted normal anatomical structures.

Important differential diagnosis of the case such as Rheumatoid Arthritis, Gouty arthritis, psoriatic arthritis, multicentric reticulohystiocytosis were excluded by absence of inflammatory pattern of illness, negative serology (RAF, anti CCP, normal uric acid level), and absence of any erosive changes on X-ray.

Patient was initially given intralesional steroids (Triamcinolone acetate 40 mg each joint) in all PIP swellings, but did not cause any reduction in six weeks period, hence was treated with surgical excision of swellings of PIP joints initially and MCP joints of bilateral hands consequently, that lead to improved hand grip and yielded complete flexion of the fingers. In addition, he was started on lipid lowering agent for dyslipidemia, and called for routine follow up.

## DISCUSSION

Although the etiology of Pachydermodactyly (PDD) is not fully understood. It is widely believed to be caused by precipitating factors in genetically susceptible individuals. In a systemic review 43% of PDD patients had history of excessive mechanical stimulation of finger joints.^4^ In addition, hormonal factors could have a role.^5^ This view is reinforced by the prevalence of PDD in young males with symptoms developing at puberty.

These swellings are believed to be caused by abnormal collagen deposition in the dermis. Swellings of PPD are characteristically limited to lateral aspect of the joint with unrestricted joint movement and no morning stiffness. Typically, PDD is painless however, a review revealed that some patients complained of pain or discomfort in their affected finger.^6^ Overlying skin may be thick or hyper pigmented.

The most common presentation of PPD is Rheumatoid like symmetrical saccular swelling involving the 2^nd^, 3^rd^ and 4^th^ proximal interphalangeal joints.^4^ Although, asymmetrical occurrence and single joint involvement is rare, but is reported.^7^

A diagnostic criterion proposed by Chen et al includes six factors: symptom less patient, no morning stiffness, no limitation to movement, swelling on lateral aspect of finger, unremarkable laboratory tests and soft tissue swelling on radiography.^8^

PDD associated with TSC has been recognized as a type; only two cases have been reported thus far in literature both occurring in 1998^6^ TSC is a benign disorder caused by mutation of tumor suppressor gene which leads to the development of multiorgan hamartomas. It usually presents with skin, neurological and behavioral manifestations. Our patient is a known case of TSC for last 3-4 years. His examination revealed a classic TSC skin lesion, angiofibroma. But there was absence of neurological or psychiatric symptoms. A thorough brain and cardiac evaluation was done to rule out any benign growths.

Since majority of PDD cases present with symptoms resulting from repeated joint trauma, cessation of stimulant activities is adequate to cause regression or stabilization. Intralesional corticosteroid has also been shown to improve swelling in some cases.^9^ While in others it is less fruitful. In our case in whom intralesional triamcinolone acetate was given for a month but it did not yield the desired outcome and ultimately surgery was opted. Surgery to improve the appearance of digit is quite common in PDD cases.^10^

## CONCLUSION

PDD is quite rare and, because of its benign and asymptomatic nature, it is often misdiagnosed. Early recognition of this disease by rheumatologist or dermatologist can prove to be beneficial for the patients. This can help minimize the long term sequel of the disease as well as help avoid unnecessary DMARD therapy, along with various trial based drug administration.

### Authors’ Contribution:

**HT and AI:** Data collection, manuscript writing.

**AK:** Wrote the manuscript and responsible for the integrity and accuracy of the study.

**RS:** Revision of the final draft.
